# Machine learning-based mortality prediction models for emergency department patients: a comparative analysis

**DOI:** 10.3389/fmed.2026.1721101

**Published:** 2026-01-30

**Authors:** Zhen Jiang, Jin Ma, Zhiqiang Guo, Qiupeng Feng, Hua Yuan

**Affiliations:** Department of Emergency Medicine, Affiliated Kunshan Hospital of Jiangsu University, Kunshan, China

**Keywords:** emergency department, LightGBM, machine learning, mortality prediction, risk stratification, SHAP analysis

## Abstract

**Background:**

Accurate mortality prediction in emergency departments (ED) is crucial for timely intervention and resource allocation. This study developed and compared multiple machine learning models to predict in-hospital mortality among ED patients.

**Methods:**

We retrospectively analyzed 1,389 ED patients admitted to Affiliated Kunshan Hospital of Jiangsu University between January and December 2021. After excluding patients under 16 years and those transferred or discharged against medical advice, we collected demographic data, vital signs, and laboratory results within 30 min of ED arrival. Nine machine learning models including Logistic Regression, Random Forest, XGBoost, LightGBM, Gradient Boosting, Support Vector Machine (SVM), Neural Network, AdaBoost, and an ensemble voting classifier were developed and compared using metrics including area under the receiver operating characteristic curve (AUROC), sensitivity, specificity, and calibration.

**Results:**

Among 1,389 patients (mean age 67.72 ± 19.28 years, 63.1% male), the mortality rate was 11.59%. LightGBM demonstrated the best performance with an AUROC of 0.9605 (95% CI: 0.94–0.98), sensitivity of 78.12%, and specificity of 93.90%. The ensemble voting classifier achieved comparable performance (AUROC: 0.9599). SHAP analysis identified serum lactate (importance: 0.252), Glasgow Coma Scale (GCS) (0.085), albumin (0.075), base excess (BE) (0.061), and systolic blood pressure (SBP) (0.049) as the top five predictive features. Calibration curves demonstrated excellent agreement between predicted and observed mortality rates, and decision curve analysis confirmed clinical utility across various threshold probabilities. Risk stratification based on predicted mortality probabilities effectively separated patients into prognostically distinct groups.

**Conclusion:**

Machine learning models, particularly LightGBM, provide highly accurate mortality prediction for ED patients. The integration of readily available clinical and laboratory parameters enables early risk stratification and may facilitate targeted interventions to improve patient outcomes.

## 1 Introduction

Emergency departments serve as critical entry points for acutely ill patients, where rapid assessment and appropriate triage decisions can significantly impact patient outcomes. Accurate early prediction of mortality risk is essential for optimal resource allocation, treatment prioritization, and informed clinical decision-making (1, 2). Traditional scoring systems such as the Modified Early Warning Score (MEWS) and the National Early Warning Score (NEWS) have been widely used in emergency settings; however, these tools often rely on limited variables and may not capture the complex, non-linear relationships between multiple clinical parameters and patient outcomes (3–7).

The advent of machine learning (ML) techniques has revolutionized predictive modeling in healthcare by enabling the analysis of high-dimensional data and the identification of complex patterns that may be imperceptible to traditional statistical methods (8–10). Recent studies have demonstrated the superiority of ML algorithms over conventional scoring systems in predicting various clinical outcomes, including mortality, sepsis, and acute kidney injury (11–15). Tree-based ensemble methods, such as Random Forest, XGBoost, and LightGBM, have shown particular promise due to their ability to handle non-linear relationships, interactions between variables, and missing data while maintaining interpretability through feature importance analysis (13, 14, 16–18).

Despite the growing body of literature on ML applications in emergency medicine, several gaps remain. First, most existing studies focus on specific patient populations or disease conditions, limiting their generalizability to the heterogeneous ED setting. Second, there is a lack of comprehensive comparison of multiple state-of-the-art ML algorithms using standardized evaluation metrics. Third, the interpretability of ML models—a critical requirement for clinical adoption—has not been adequately addressed through modern explainable AI techniques such as SHapley Additive exPlanations (SHAP) (19–21).

Motivated by these gaps, we developed a reproducible modeling pipeline using early ED variables available within 30 min of arrival (demographics, vital signs, GCS, and arterial blood gas–related laboratory markers) and performed a head-to-head comparison of nine common ML approaches, including gradient-boosting frameworks (XGBoost, LightGBM) and an ensemble soft-voting classifier. We evaluated each model across discrimination, calibration, and decision-analytic utility, and used SHAP to improve interpretability of the top-performing model.

Accordingly, our objectives were to: (1) identify the optimal ML algorithm for in-hospital mortality prediction in an unselected ED cohort; (2) determine the most important predictive features; (3) evaluate model calibration and clinical utility; and (4) provide an interpretable and reproducible framework that can support future external validation and implementation.

## 2 Methods

### 2.1 Study design and setting

This retrospective cohort study was conducted at the Emergency Department of Affiliated Kunshan Hospital of Jiangsu University, a tertiary care hospital in Jiangsu Province, China. The study was approved by the Ethics Review Committee of Kunshan Affiliated Hospital of Jiangsu University (No. 2024–03-002-K01) and conducted in accordance with the Declaration of Helsinki. The requirement for informed consent was waived due to the retrospective nature of the study.

### 2.2 Study population and data collection

We included all patients aged 16 years or older who presented to the ED between January 1, 2021, and December 31, 2021. Exclusion criteria comprised: (1) patients under 16 years of age; (2) patients transferred to other institutions; and (3) patients who left against medical advice. The primary outcome was in-hospital mortality (all-cause death during hospitalization).

We collected comprehensive clinical data including demographic parameters (age and gender), vital signs measured within 30 min of ED arrival (systolic blood pressure [SBP], heart rate, respiratory rate, body temperature, and peripheral oxygen saturation [SpO2]), Glasgow Coma Scale (GCS) score, and laboratory results from arterial blood gas analysis. Blood samples were obtained within 30 min of ED arrival and analyzed using the ABL90 FLEX blood gas analyzer (Radiometer Medical ApS, Denmark). Laboratory parameters included pH, serum lactate, base excess (BE), hemoglobin (HB), white blood cell count (WBC), lymphocyte count, platelet count, glucose, albumin, prothrombin time (PT), and activated partial thromboplastin time (APTT).

### 2.3 Data preprocessing

All records with missing values in any variable were excluded from the analysis to ensure data completeness and model reliability. The final cleaned dataset comprised 1,389 patients with complete data for all variables.

### 2.4 Model development and validation

We randomly split the dataset into training (80%, *n* = 1,111) and testing (20%, *n* = 278) sets using stratified sampling to maintain the original outcome distribution. For models requiring scaled inputs (Logistic Regression, SVM, and Neural Network), we applied standardization using StandardScaler to normalize features to zero mean and unit variance, fitted on the training set and applied to the testing set to prevent data leakage.

To enable reproducibility, all splits and model training were performed using a fixed random seed (random_state = 42) in scikit-learn and equivalent seed settings in XGBoost/LightGBM where applicable.

Nine ML algorithms were developed and compared: (1) Logistic Regression with L2 regularization; (2) Random Forest with 200 trees; (3) eXtreme Gradient Boosting (XGBoost); (4) Light Gradient Boosting Machine (LightGBM); (5) Gradient Boosting; (6) Support Vector Machine with radial basis function kernel; (7) Multi-layer Perceptron Neural Network with three hidden layers (100, 50, 25 neurons); (8) Adaptive Boosting (AdaBoost); and (9) an ensemble voting classifier combining the top-performing models using soft voting. All tree-based models incorporated class balancing to address the imbalanced nature of mortality outcomes. Hyperparameters were optimized through grid search with 5-fold stratified cross-validation on the training set.

### 2.5 Model evaluation

Model performance was assessed using multiple metrics. Discrimination was evaluated using the area under the receiver operating characteristic curve (AUROC), area under the precision-recall curve (AUPRC), sensitivity, specificity, positive predictive value (precision), F1-score, and Brier score.

Cross-validation performance was assessed using 5-fold stratified cross-validation to evaluate model stability and generalizability. Learning curves were generated for the best-performing model to assess the relationship between training set size and model performance.

Risk stratification was performed by categorizing patients into four risk groups based on predicted mortality probability: low risk (0–25%), medium risk (25–50%), high risk (50–75%), and very high risk (75–100%). We compared actual mortality rates across these risk strata.

### 2.6 Feature importance and model interpretability

Feature importance was quantified for all models supporting this functionality. For tree-based models, we used built-in feature importance scores based on information gain. We calculated mean feature importance across all models to identify the most consistently important predictors.

To enhance model interpretability, we applied SHAP analysis to the best-performing model. SHAP values quantify each feature’s contribution to individual predictions using game-theoretic principles, providing both global feature importance and local explanations for individual predictions. This allows clinicians to understand not only which features are globally important, but also how specific values of key variables (e.g., a particular lactate level or GCS score) contribute to a given patient’s predicted mortality risk.

### 2.7 Statistical analysis

Descriptive statistics are presented as mean ± standard deviation and median [minimum-maximum] for continuous variables, and as frequency (percentage) for categorical variables. All analyses were performed using Python 3.10 with scikit-learn 1.3, XGBoost 2.0, LightGBM 4.1, CatBoost 1.2, and SHAP 0.43 libraries. Statistical significance was defined as *p* < 0.05.

## 3 Results

### 3.1 Patient characteristics

After applying exclusion criteria and removing cases with missing data, the final cohort comprised 1,389 patients. The mean age was 67.72 ± 19.28 years (median: 73 years, range: 16–104 years), with males comprising 63.1% (*n* = 876) of the cohort. The overall in-hospital mortality rate was 11.59% (161 deaths). Baseline characteristics are summarized in Table 1. The mean GCS score was 13.72 ± 2.67, indicating generally preserved consciousness across the cohort. Mean vital signs included SBP 141.28 ± 32.47 mmHg, heart rate 100.16 ± 25.28 bpm, respiratory rate 23.40 ± 5.59 breaths/min, temperature 37.21 ± 0.94 °C, and SpO2 90.96 ± 10.24%. Laboratory results showed mean serum lactate of 2.64 ± 3.15 mmol/L, pH 7.36 ± 0.13, BE −1.21 ± 8.18 mmol/L, hemoglobin 131.43 ± 27.77 g/L, WBC 11.16 ± 7.34 × 110^9^/L, lymphocyte count 1.67 ± 3.25 × 10^9^/L, platelet count 202.96 ± 86.16 × 10^9^/L, glucose 11.14 ± 7.80 mmol/L, albumin 39.97 ± 11.00 g/L, PT 12.75 ± 4.64 s, and APTT 31.11 ± 7.20 s. To further characterize the heterogeneity of the study population, baseline variables stratified by survival status (survivors vs. non-survivors) are provided in Supplementary Table S1.

**Table 1:** Descriptive statistics for emergency department patients.

Variable	Mean±SD/ *N* (%)	Median [Min-Max]
Gender		
Male	876 (63.1%)	–
Female	513 (36.9%)	–
Age	67.72 ± 19.28	73.00 [16.00–104.00]
GCS	13.72 ± 2.67	15.00 [3.00–15.00]
Temperature	37.21 ± 0.94	37.00 [33.60–40.50]
Heart rate	100.16 ± 25.28	99.00 [10.00–212.00]
Respiratory rate	23.40 ± 5.59	22.00 [0.00–49.00]
SBP	141.28 ± 32.47	140.00 [43.00–265.00]
SpO_2_	90.96 ± 10.24	95.00 [39.00–100.00]
PH	7.36 ± 0.13	7.39 [6.77–7.67]
Serum lactate	2.64 ± 3.15	1.60 [0.20–26.00]
BE	−1.21 ± 8.18	−0.40 [−29.50–27.00]
HB	131.43 ± 27.77	133.00 [34.00–225.00]
WBC	11.16 ± 7.34	9.80 [0.48–151.87]
Lymphocyte	1.67 ± 3.25	1.14 [0.09–103.00]
Platelet	202.96 ± 86.16	195.00 [6.00–725.00]
Glucose	11.14 ± 7.80	8.60 [0.90–73.30]
Albumin	39.97 ± 11.00	40.30 [16.10–377.00]
PT	12.75 ± 4.64	11.90 [1.20–104.20]
APTT	31.11 ± 7.20	30.20 [0.99–106.60]

### 3.2 Distribution analysis

**Figure 1:**
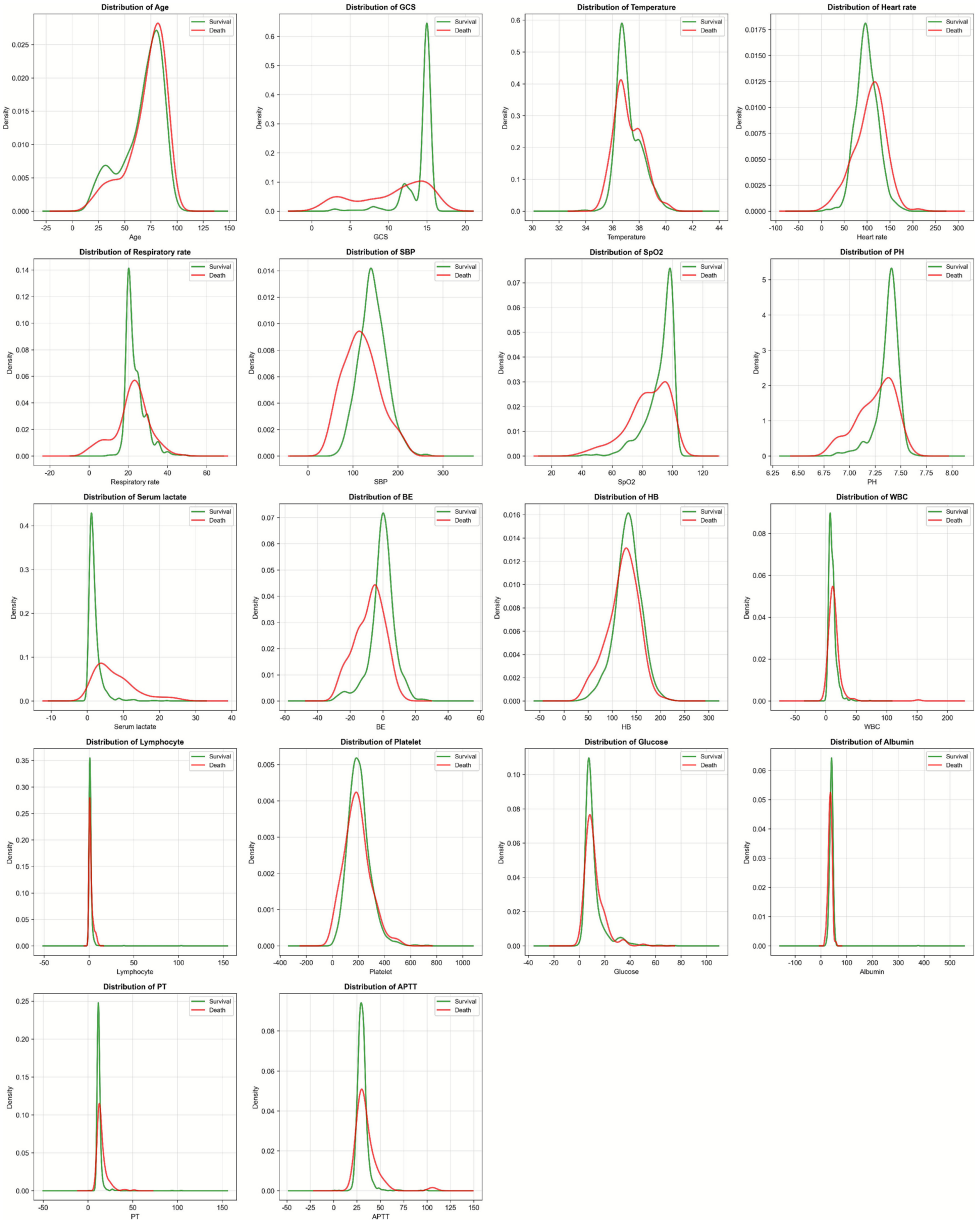
Variable distributions using kernel density estimation plots.

Kernel density estimation plots revealed distinct distributional differences between survivors and non-survivors across multiple variables. Non-survivors exhibited higher serum lactate levels, lower GCS scores, more negative BE values, and lower SBP compared to survivors (Figure 1).

### 3.3 Model performance comparison

**Figure 2:**
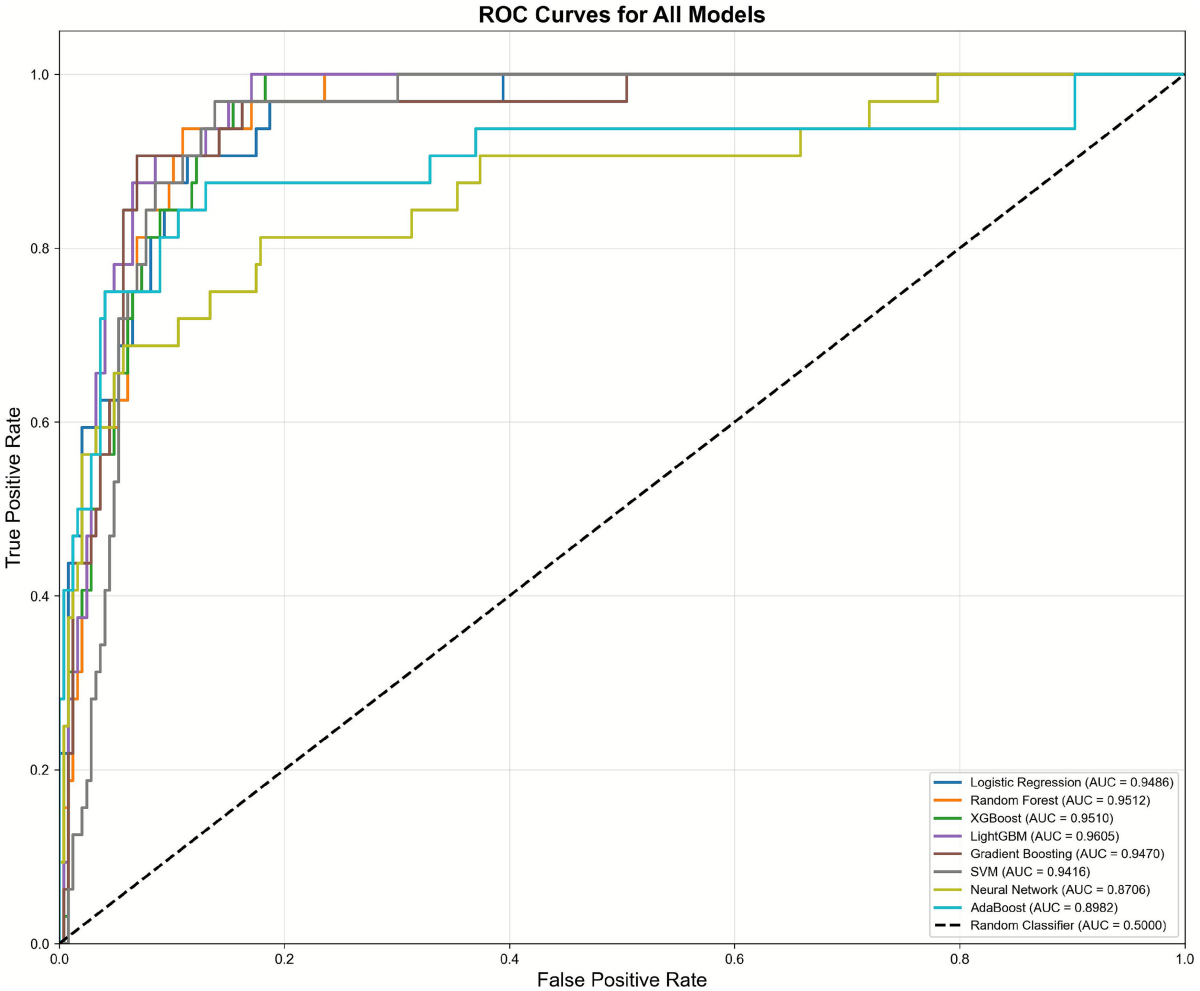
ROC curves of different individual machine learning models.

All nine models demonstrated excellent discrimination ability with AUROC values exceeding 0.87 (Table 2). LightGBM achieved the highest AUROC of 0.9605 (95% CI: 0.94–0.98), followed closely by the ensemble voting classifier (0.9599) and Random Forest (0.9512). LightGBM also demonstrated optimal balance across multiple metrics with accuracy of 92.09%, precision of 62.50%, sensitivity of 78.12%, specificity of 93.90%, F1-score of 69.44%, AUPRC of 0.6769, and Brier score of 0.0596, indicating excellent calibration. The ROC curves demonstrated superior performance of tree-based ensemble methods compared to traditional approaches. LightGBM, ensemble voting, and Random Forest showed nearly identical ROC curves with minimal separation from the upper-left corner (Figure 2). From a clinical perspective, this level of discrimination suggests that these models can reliably distinguish patients at low versus high risk of in-hospital mortality based on information available very early in the ED stay.

**Table 2:** Single-model and ensemble performance on the test set.

Model	Accuracy	Precision	Recall	Specificity	F1-Score	ROC-AUC	PR-AUC	Brier score
Logistic Regression	0.8777	0.4833	0.9062	0.874	0.6304	0.9486	0.7417	0.1014
Random Forest	0.9209	0.6786	0.5938	0.9634	0.6333	0.9512	0.6422	0.0592
XGBoost	0.9137	0.6053	0.7188	0.939	0.6571	0.951	0.6327	0.069
LightGBM	0.9209	0.625	0.7812	0.939	0.6944	0.9605	0.6769	0.0596
Gradient Boosting	0.9137	0.625	0.625	0.9512	0.625	0.947	0.653	0.0613
SVM	0.9029	0.549	0.875	0.9065	0.6747	0.9416	0.5441	0.0662
Neural Network	0.8993	0.8333	0.1562	0.9959	0.2632	0.8706	0.6457	0.0698
AdaBoost	0.9209	0.6316	0.75	0.9431	0.6857	0.8982	0.7367	0.2252
Ensemble (Voting)	0.9317	0.6757	0.7812	0.9512	0.7246	0.9599	0.6927	0.0571

### 3.4 Cross-validation results

Five-fold stratified cross-validation confirmed the robustness of the models, with mean cross-validation AUROC values ranging from 0.8627 (AdaBoost) to 0.9241 (Random Forest). LightGBM achieved a mean cross-validation AUROC of 0.9070 ± 0.0382, demonstrating stable performance across different data subsets with minimal overfitting (Table 3). The learning curve for LightGBM showed convergence of training and validation scores, indicating adequate sample size and minimal overfitting.

**Table 3:** Five-fold stratified cross-validation results.

Model	Mean ROCAUC	Std	Min	Max
Logistic regression	0.9074	0.0398	0.8607	0.9653
Random forest	0.9241	0.0309	0.8777	0.9678
XGBoost	0.9157	0.0367	0.862	0.9631
LightGBM	0.907	0.0382	0.8709	0.969
Gradient boosting	0.9122	0.0255	0.8711	0.9392
SVM	0.9108	0.0318	0.8721	0.9525
Neural network	0.8885	0.0273	0.8605	0.9389
AdaBoost	0.8627	0.0479	0.8073	0.9331

### 3.5 Feature importance analysis

**Figure 3:**
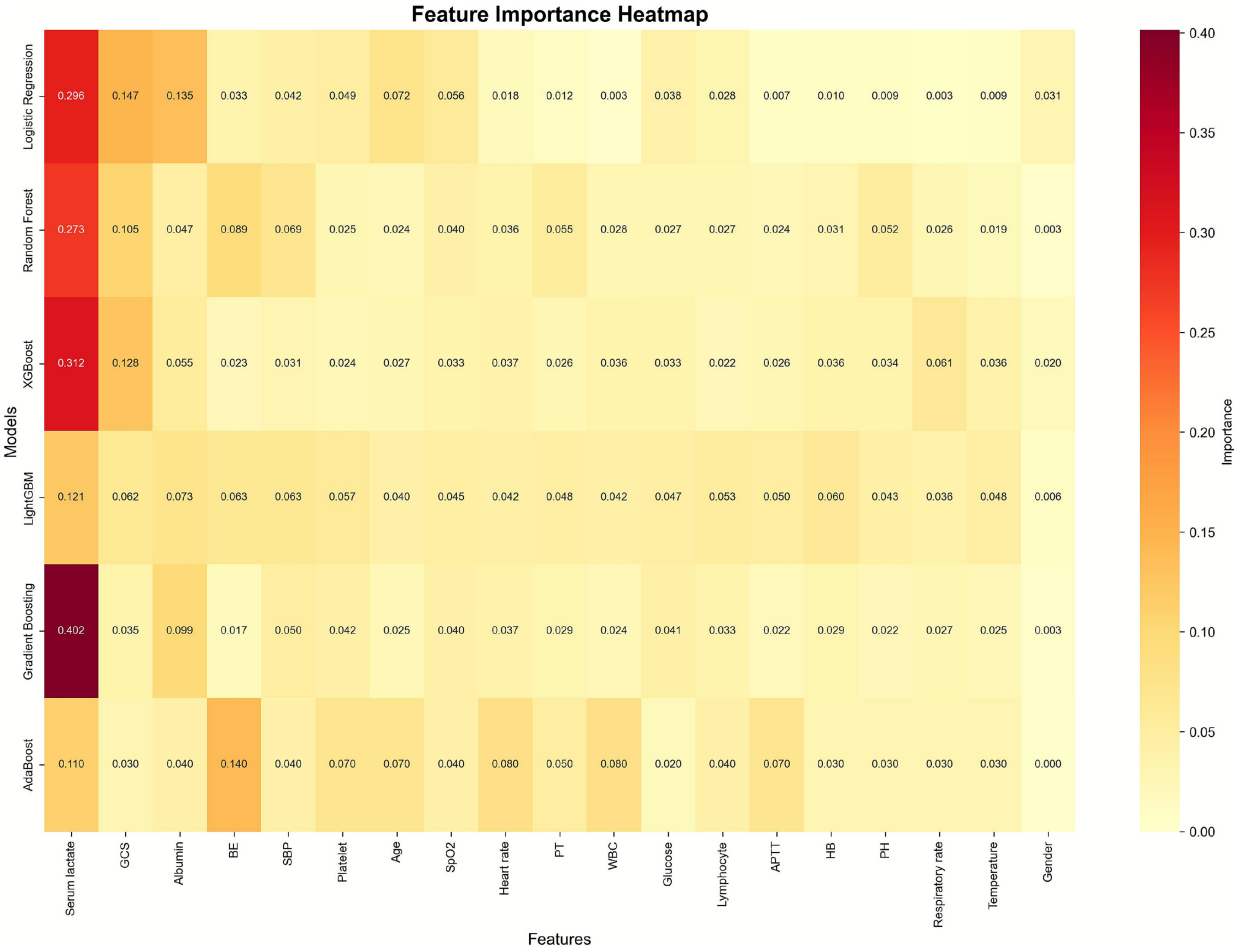
The importance of features in mortality prediction using heatmap.

Feature importance analysis identified serum lactate as the most important predictor across all models (mean importance: 0.252), followed by GCS (0.085), albumin (0.075), BE (0.061), and SBP (0.049) (Table 4; Figure 3). These findings were consistent across different model architectures, lending credibility to their biological and clinical significance. Notably, gender showed minimal predictive importance (0.011), suggesting that mortality risk in the ED is primarily driven by acute physiological derangements rather than demographic factors.

**Table 4:** Feature importance.

Feature	Logistic regression	Random forest	XGBoost	LightGBM	Gradient boosting	AdaBoost	Mean
Serum lactate	0.296	0.273	0.312	0.121	0.402	0.110	0.252
GCS	0.147	0.105	0.128	0.062	0.035	0.030	0.085
Albumin	0.135	0.047	0.055	0.073	0.099	0.040	0.075
BE	0.033	0.089	0.023	0.063	0.017	0.140	0.061
SBP	0.042	0.069	0.031	0.063	0.050	0.040	0.049
Platelet	0.049	0.025	0.024	0.057	0.042	0.070	0.044
Age	0.072	0.024	0.027	0.040	0.025	0.070	0.043
SpO2	0.056	0.040	0.033	0.045	0.040	0.040	0.042
Heart rate	0.018	0.036	0.037	0.042	0.037	0.080	0.042
PT	0.012	0.055	0.026	0.048	0.029	0.050	0.037
WBC	0.003	0.028	0.036	0.042	0.024	0.080	0.036
Glucose	0.038	0.027	0.033	0.047	0.041	0.020	0.034
Lymphocyte	0.028	0.027	0.022	0.053	0.033	0.040	0.034
APTT	0.007	0.024	0.026	0.050	0.022	0.070	0.033
HB	0.010	0.031	0.036	0.060	0.029	0.030	0.033
PH	0.009	0.052	0.034	0.043	0.022	0.030	0.032
Respiratory rate	0.003	0.026	0.061	0.036	0.027	0.030	0.031
Temperature	0.009	0.019	0.036	0.048	0.025	0.030	0.028
Gender	0.031	0.003	0.020	0.006	0.003	0.000	0.011

### 3.6 SHAP analysis and model interpretability

**Figure 4:**
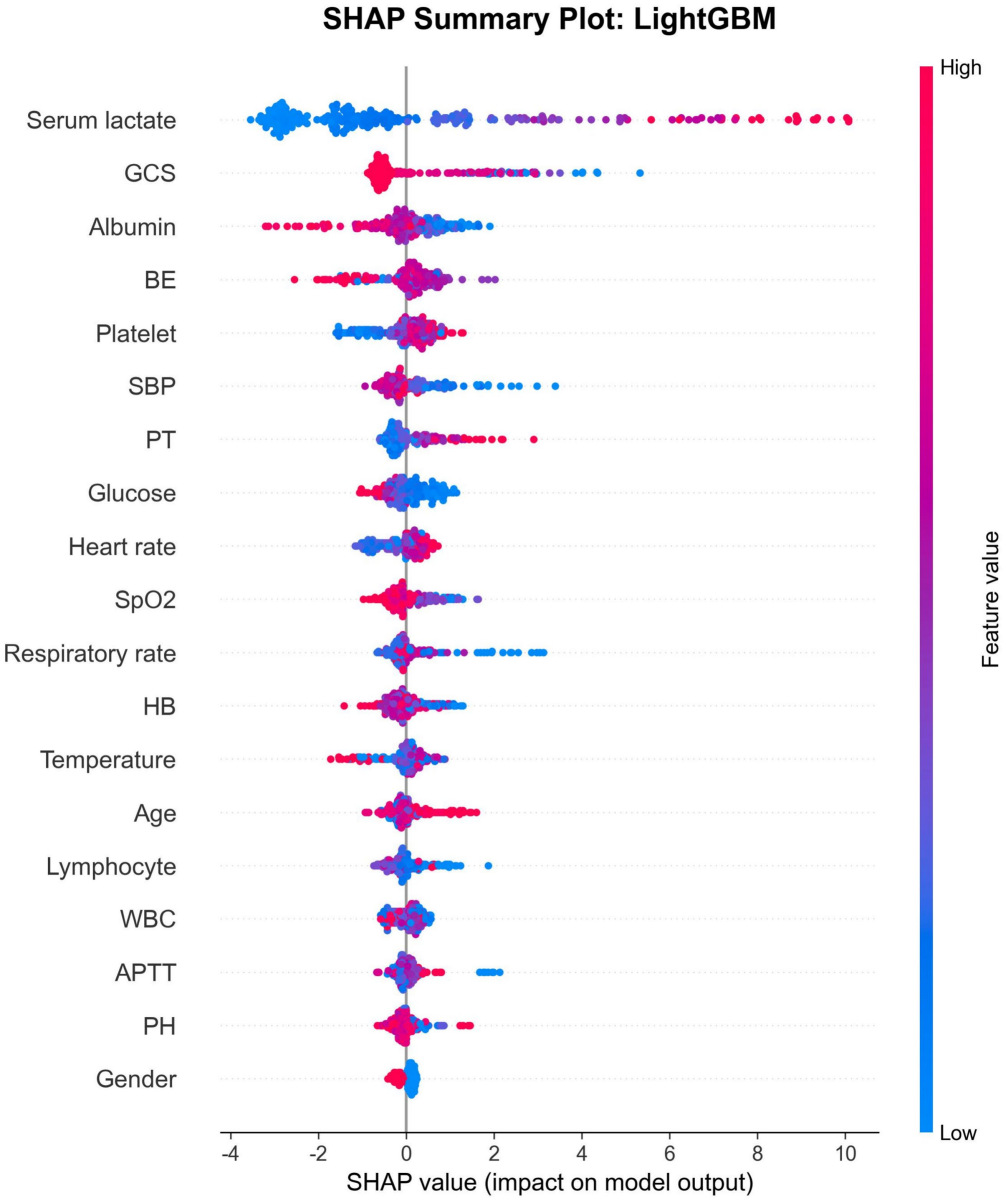
The SHAP summary plot of LightGBM model.

**Figure 5:**
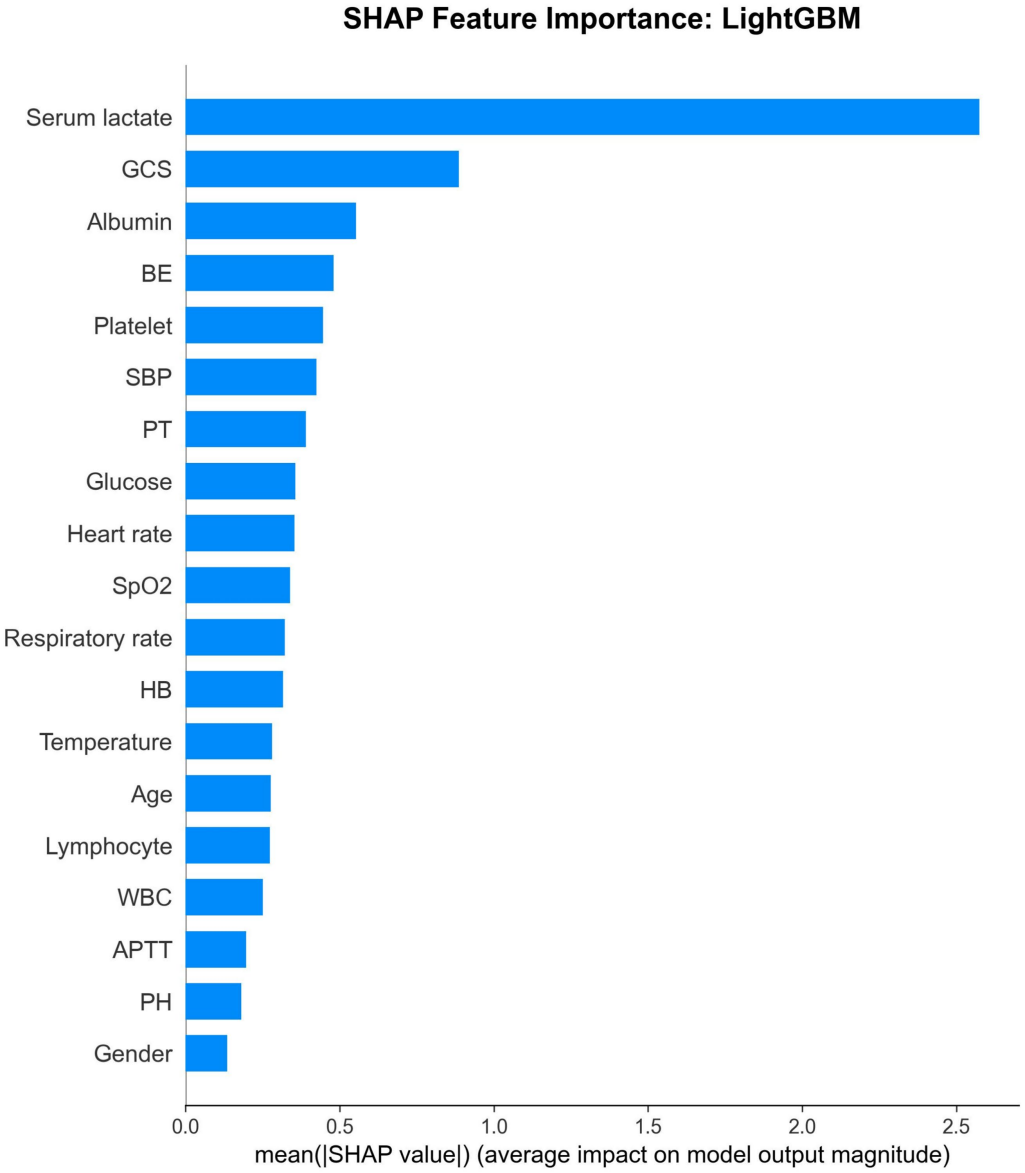
Feature importance ranking using SHAP.

SHAP analysis of the best-performing LightGBM modelprovided detailed insights into feature contributions. The SHAP summary plot demonstrated that higher serum lactate values consistently increased mortality risk, while higher GCS scores decreased risk (Figures 4 and 5). The SHAP feature importance plot confirmed serum lactate, GCS, and albumin as the three most influential features.

### 3.7 Risk stratification

Risk stratification analysis demonstrated effective separation of patients into distinct prognostic groups. The very high-risk group (predicted probability >75%, *n* = 34) had an observed mortality rate of 70.59%, substantially exceeding the cohort average of 11.59% (Table 5). The high-risk group (50–75%, *n* = 6) showed 16.67% mortality, the medium-risk group (25–50%, *n* = 3) showed 66.67% mortality, and the low-risk group (<25%, *n* = 235) showed only 2.13% mortality. However, the medium- and high-risk strata contained very few patients in the test set (*n* = 3 and *n* = 6, respectively). In contrast, the low- and very high-risk groups were larger and showed a clear and clinically meaningful gradient in observed mortality, suggesting that the model is most robust for distinguishing patients at the extremes of predicted risk.

**Table 5:** The actual mortality rate of the risk score constructed by the model in the test set.

Risk group	*N* (%)
Low risk	235 (2.13%)
Medium risk	3 (66.67%)
High risk	6 (16.67%)
Very high risk	34 (70.59%)

## 4 Discussion

This comprehensive study compared nine machine learning algorithms for predicting in-hospital mortality among ED patients and demonstrated that tree-based ensemble methods, particularly LightGBM, achieve excellent discrimination and clinical utility. Our findings provide a framework for implementing interpretable, clinically useful prediction models.

The AUROC of 0.9605 achieved by LightGBM substantially exceeds the performance of traditional severity scoring systems. A meta-analysis by Patel et al. reported pooled AUROCs of 0.5–0.89 for EWS in predicting in-hospital mortality (22). Our results align with recent ML studies showing superior performance: Klug et al. reported an AUROC of 0.962 using gradient boosting on a large Israeli cohort (23). The exceptional performance in our study may reflect the comprehensive feature set including laboratory parameters, which are often unavailable in studies relying solely on vital signs and demographics.

The identification of serum lactate as the most important predictor aligns with extensive literature establishing lactate as a marker of tissue hypoperfusion and anaerobic metabolism. A meta-analysis by Haas et al. (24) demonstrated that initial lactate levels >10 mmol/L are associated with increased mortality across diverse patient populations. The importance of GCS as the second-ranked predictor underscores the prognostic significance of altered consciousness. Reduced GCS reflects both primary neurological injury and secondary effects of systemic illness, serving as an integrated measure of illness severity (25, 26). The relatively low importance of demographic factors (age ranked 7th, gender 19th) suggests that acute physiological derangements dominate mortality risk in the ED setting.

Our risk stratification analysis illustrates how the model might be used in practice. Patients categorized as very high risk (>75% predicted probability) experienced markedly elevated observed mortality, suggesting that they may benefit from early intensive monitoring, expedited diagnostic work-up, and prompt consideration of ICU admission when appropriate. Conversely, patients in the low-risk group (<25% predicted probability) had very low observed mortality, which might help support safe de-escalation of monitoring intensity or ED disposition decisions when consistent with the overall clinical picture. Importantly, the model is intended as a decision-support tool rather than a replacement for clinical judgment. In some cases, repeated measurements and close observation may be more informative than a single early lactate value, even though the model recognizes lactate as a strong predictor at the population level.

This study has several limitations. First, it was a retrospective, single-center analysis conducted in a tertiary hospital in China. As such, the case mix, practice patterns, and resource availability may differ from those in other institutions or countries, and external validation in independent cohorts is required before broad implementation. Second, we excluded patients with any missing values to facilitate model development on complete cases. While this approach simplifies analysis and may improve model stability, it can introduce selection bias if patients with missing data differ systematically from those with complete information. Third, we did not include comorbidity indices, medication use, or additional laboratory tests beyond those obtained from arterial blood gas analysis, which may have limited the ability of the models to capture the full spectrum of risk factors. Finally, the numbers of patients in the medium- and high-risk strata in the test set were small, leading to unstable mortality estimates in these intermediate categories; the risk stratification is most robust at the extremes (low vs. very high risk).

## 5 Conclusion

Machine learning models, particularly LightGBM, demonstrate excellent performance for predicting in-hospital mortality among emergency department patients. Serum lactate, GCS, albumin, base excess, and systolic blood pressure emerge as the most important predictors, all readily available within 30 min of ED arrival. These findings support the potential for implementing ML-based decision support systems to enhance early risk assessment in emergency departments.

## Data Availability

The original contributions presented in the study are included in the article/Supplementary material, further inquiries can be directed to the corresponding author/s.
