# Supplementary material for: Machine learning-based mortality prediction models for emergency department patients: a comparative analysis
Source: Front Med (Lausanne). 2026 Jan 30;13:1721101. doi: 10.3389/fmed.2026.1721101 (PMC12901469; doi:10.3389/fmed.2026.1721101)
Supplement: Supplementary file 1 [file Data_Sheet_1.pdf]

Supplementary Tables 1. The differences in baseline characteristics between participants in the survival group and the death group.

Variable	Survivors	Non survivors	P value
Gender			0.969
Male	777 (63.1%)	102 (63.0%)	
Female	454 (36.9%)	60 (37.0%)	
Age	67.21 ± 19.42	71.52 ± 17.90	0.005
APTT	30.64 ± 6.01	34.70 ± 12.52	<0.001
Albumin	40.51 ± 11.28	35.77 ± 7.33	<0.001
BE	-0.31 ± 7.64	-8.05 ± 8.83	<0.001
GCS	14.13 ± 1.99	10.48 ± 4.48	<0.001
Glucose	11.09 ± 7.88	11.50 ± 7.15	0.498
HB	132.69 ± 27.03	121.77 ± 31.37	<0.001
Heart rate	99.47 ± 23.87	105.34 ± 33.75	0.033
Lymphocyte	1.60 ± 3.35	2.17 ± 2.24	0.004
PH	7.38 ± 0.11	7.26 ± 0.19	<0.001
PT	12.42 ± 4.35	15.36 ± 5.83	<0.001
Platelet	204.68 ± 84.28	191.57 ± 99.90	0.112
Respiratory rate	23.60 ± 4.99	21.86 ± 8.76	0.014
SBP	144.16 ± 30.24	119.38 ± 39.57	<0.001
Serum lactate	1.99 ± 1.88	7.56 ± 5.59	<0.001
SpO2	91.94 ± 9.25	83.59 ± 13.76	<0.001
Temperature	37.22 ± 0.92	37.19 ± 1.03	0.712
WBC	10.80 ± 6.14	13.83 ± 13.01	0.004
